# Gastrointestinal digestibility insights of different levels of coated complex trace minerals supplementation on growth performance of yellow-feathered broilers

**DOI:** 10.3389/fvets.2022.982699

**Published:** 2022-09-13

**Authors:** Chuanbin Chen, Mingren Qu, Huan Liang, Kehui Ouyang, Zhihui Xiong, Youchang Zheng, Qiuliang Yan, Lanjiao Xu

**Affiliations:** ^1^Jiangxi Province Key Laboratory of Animal Nutrition, Animal Nutrition and Feed Safety Innovation Team, Jiangxi Agricultural University, Nanchang, China; ^2^Gongqingcheng Element Animal Nutrition Co., Ltd., Gongqingcheng, China; ^3^Jilin Academy of Agricultural Sciences, Changchun, China

**Keywords:** yellow-feathered broilers, coated complex trace minerals, growth performance, nutrient digestibility, intestinal development

## Abstract

This study was designed to evaluate the optimum additional level of coated complex trace minerals (TMs) and its impacts on the growth performance of broilers through measurement of digestibility of nutrients and intestinal development. In a 56-day trial, a total of 360 one-day-old male yellow-feathered broilers were randomly divided into six dietary treatment groups. Each treatment contained six replicates, with 10 birds. The control group was supplemented with 1,000 mg/kg of uncoated complex TMs in the basal diet (UCCTM1000). The remaining 5 treatments were degressively supplemented with coated complex TMs from 1,000 to 200 mg/kg in the basal diet, which were considered as (CCTM1000), (CCTM800), (CCTM600), (CCTM400), (CCTM200), respectively. Results: On comparing the UCCTM1000 supplementation, the CCTM1000 supplementation decreased the feed to gain ratio (F/G) (*P* < 0.05), increased digestibility of crude protein (CP) (*P* < 0.05), crude fat (CF) (*P* < 0.05), villus height (VH) of duodenum (*P* < 0.05), and the mRNA expression level of occludin in jejunal mucosa (*P* < 0.05). In addition, the F/G was lower in the CCTE600 group than that in the CCTE200 group (*P* < 0.05). The VH to crypt depth (CD) ratio (V/C) of jejunum and ileum in the CCTM400 and CCTM600 groups was higher (*P* < 0.05) than that in the CCTM1000 group. The serum endotoxin and D-lactate level and CP digestibility were increased by dietary coated complex TMs addition level. The mRNA expression levels of claudin-1 and ZO-1 in the CCTM600 group were higher (*P* < 0.05) than that in the CCTM1000 group. In conclusion, adding 600 mg/kg of coated complex TMs showed the minimum F/G and the maximum crude protein digestibility and intestine development of yellow-feathered broilers compared with other treatments. This supplementation level of coated complex TMs could totally replace 1,000 mg/kg of uncoated complex TMs to further decrease the dose of TMs and raise economic benefit.

## Introduction

Trace minerals, mainly copper, iron, manganese, zinc, and selenium, perform a key role in the growth, nutritional regulation, and intestinal health of the poultry by participating in numerous biochemical pathways (1–3). Trace minerals (TMs) influence appetite, growth performance, bone growth, and feathering of poultry (4). The minerals deficiency may result in loss of appetite, growth retardation, anemia, etc., in animals (5–7). Appropriate using of TMs additives can make up for the deficiency of some minerals in diets and improve the performance of poultry (8, 9). Traditionally, inorganic TMs are added to broilers' diets to maintain levels of minerals that enable broilers to reach their growth potential (10–12). Inorganic TMs have many problems, such as unstable properties, damage to some nutrients (such as vitamins, amino acids), and poor palatability (13). In commercial poultry production, inorganic TMs are used to supply from two to ten times more of these minerals than NRC recommendations (14). Excessive use of inorganic salts causes damage to nutrient absorption and low mineral bioavailability (15). In addition, current excessive mineral intake causes environmental pollution by higher heavy mineral excretion (16). Excessive use of feces with high mineral concentration was used as fertilizer; it will lead to high mineral concentration in the soil thereby decreasing crop yield (17) with remaining minerals penetrating into the soil and may pollute under water supplies (18). Therefore, in order to overcome the deficiency in the use of inorganic trace elements, coated TMs may be used as an alternative choice.

Coating technology has been widely used in the field of feed additives. The so-called coating refers to the material that can form a protective film on the surface of solid drugs in a specific instrument and according to a specific process, after drying, it forms a kind of protective layer that adheres closely to the surface (19). Coated TMs have the characteristics of a small impact on the destruction of vitamins and amino acids in the feed, uniform particles, good dispersibility, high mixing uniformity, and less dust. It has been found that supplementation with low concentrations of coated zinc oxide and high concentrations of zinc oxide had the same effect on intestinal immunity defense systems in weaned piglets (20). At present, there are few reports on the application of coating technology-based complex TMs in poultry production. Therefore, this study was designed to optimize the level of coated complex TMs and their impacts on the growth performance of yellow-feathered broilers through the measurement of digestibility of nutrients and intestinal development.

## Materials and methods

### Ethical approval

This study was approved and carried out under the Institutional Animal Care and Use Committee of Jiangxi Agricultural University (Nanchang, Jiangxi, China). The number of the ethics committee-approved protocols for the animal study was JXAULL-2019-16.

### Animals and experimental design

In a 56- day trial, a total of 360 one-day-old (male, BW: 32.61 ± 0.06 g) yellow-feathered broilers was randomly divided into 6 dietary treatment groups. Each treatment contained 6 replicates, with 10 birds. The control group supplemented with 1,000 mg/kg of uncoated complex TMs (a basal diet pules copper chloride, ferrous sulfate, zinc sulfate, manganese sulfate, sodium selenite, and calcium iodate providing commercially utilized levels in China of 10, 105, 100, 98, 0.9, and 0.575 mg/kg, respectively) was considered as UCCTM1000. The remaining five treatments were degressively supplemented with coated complex TMs from 1,000 to 200 mg/kg in the basal diet, which were considered as (CCTM1000), (CCTM800), (CCTM600), (CCTM400), (CCTM200), respectively. The uncoated complex TMs and coated complex TMs used in the experiment were provided by Gongqingcheng Element Animal Nutrition Co., Ltd. (Gongqingcheng, Jiangxi, China). Supplementary complex inorganic TMs in the UCCTM1000 group and CCTM1000 group diets were formulated to be typical of those currently used in the Chinese broiler chickens industry. Dietary treatments varied in TMs composition are shown in Table 1. Both coated and uncoated minerals were thoroughly mixed and homogenized with premix, respectively, and then blended in a mixer with soya bean. The composition and nutrient levels of the experimental diets in two stages are shown in as shown in Tables 2, 3.

**Table 1:** Supplemental levels of trace minerals (TMs) in experimental diets (mg/kg).

**Item^b^**	**Experimental treatments** ^**a**^					
	**UCCTM1000**	**CCTM1000**	**CCTE800**	**CCTE600**	**CCTE400**	**CCTE200**
Cu	10	10	8	6	4	2
Fe	105	105	84	63	42	21
Zn	100	100	80	60	40	20
Mn	98	98	78.4	58.8	39.2	19.6
I	0.9	0.9	0.72	0.54	0.36	0.18
Se	0.575	0.575	0.46	0.345	0.23	0.115

^a^UCCTM1000, supplemented with 1,000 mg/kg of uncoated complex trace minerals; CCTE1000, supplemented with 1,000 mg/kg of coated complex trace minerals; CCTE800, supplemented with 800 mg/kg of coated complex trace minerals; CCTE600, supplemented with 600 mg/kg of coated complex trace minerals; CCTE400, supplemented with 400 mg/kg of coated complex trace minerals; CCTE200, supplemented with 200 mg/kg of coated complex trace minerals.

^b^Raw material composition: a basal diet pules copper chloride, ferrous sulfate, zinc sulfate, manganese sulfate, sodium selenite, and calcium iodate providing commercially utilized levels in China of 10, 105, 100, 98, 0.9, and 0.575 mg/kg, respectively.

**Table 2:** Composition and nutrient levels of the diets for the starter period (1–28 days).

**Items**	**Uncoated**	**Coated**			
**Level (mg/kg)**	**1,000**	**1,000**	**800**	**600**	**400**	**200**
Corn	58.88	58.88	58.88	58.88	58.88	58.88
Soybean meal (43%)	29.12	29.12	29.12	29.12	29.12	29.12
Rapeseed meal	5.00	5.00	5.00	5.00	5.00	5.00
Soybean oil	2.00	2.00	2.00	2.00	2.00	2.00
CaHPO_4_	1.80	1.80	1.80	1.80	1.80	1.80
Limestone	1.17	1.17	1.17	1.17	1.17	1.17
L-Lys	0.30	0.30	0.30	0.30	0.30	0.30
DL-Met	0.15	0.15	0.15	0.15	0.15	0.15
NaCl	0.30	0.30	0.30	0.30	0.30	0.30
Zeolite powder	0.18	0.18	0.20	0.22	0.24	0.26
Vitamin premix^a^	1.00	1.00	1.00	1.00	1.00	1.00
Trace minerals^b^	0.10	0.10	0.08	0.06	0.04	0.02
Total	100.00	100.00	100.00	100.00	100.00	100.00
**Calculated nutrient level**						
Metabolizable energy (MJ/kg)	12.14	12.14	12.14	12.14	12.14	12.14
Crude protein (%)	21.00	21.00	21.00	21.00	21.00	21.00
Calcium (%)	1.22	1.22	1.22	1.22	1.22	1.22
Available P (%)	0.55	0.55	0.55	0.55	0.55	0.55
Lysine (%)	1.38	1.38	1.38	1.38	1.38	1.38
Methionine (%)	0.49	0.49	0.49	0.49	0.49	0.49
Methionine + Cystine (%)	0.86	0.86	0.86	0.86	0.86	0.86

^a^The vitamin premix provided the following per kilogram of diet: vitamin A (retinyl acetate) 12,000 IU, vitamin D3 (cholecalciferol) 3,000 IU, vitamin E 20 mg, vitamin K3 1.3 mg, thiamine 2.2 mg, riboflavin 10 mg, Vitamin B3 10 mg, choline 400 mg, vitamin B5 50 mg, pyridoxine 4 mg, Biotin 0.40 mg, vitamin B11 1 mg, and vitamin B12 0.013 mg.

^b^The trace minerals provided the following per kilogram of diet: Cu 10 mg (cupric chloride), Fe 105 mg (ferrous sulfate), Zn 100 mg (Zinc sulfate), Mn 98 mg (manganous sulfate), Se 0.9 mg (sodium selenite), I (calcium iodate) 0.575 mg.

**Table 3:** Composition and nutrient levels of the diets for the grower period (29–56 days).

**Items**	**Uncoated**	**Coated**				
**Level (mg/kg)**	**1,000**	**1,000**	**800**	**600**	**400**	**200**
Corn	60.23	60.23	60.23	60.23	60.23	60.23
Soybean meal (43%)	27.41	27.41	27.41	27.41	27.41	27.41
Rapeseed meal	4.00	4.00	4.00	4.00	4.00	4.00
Soybean oil	3.36	3.36	3.36	3.36	3.36	3.36
CaHPO_4_	1.40	1.40	1.40	1.40	1.40	1.40
Limestone	1.20	1.20	1.20	1.20	1.20	1.20
L-Lys	0.35	0.35	0.35	0.35	0.35	0.35
DL-Met	0.08	0.08	0.08	0.08	0.08	0.08
NaCl	0.30	0.30	0.30	0.30	0.30	0.30
Zeolite powder	0.57	0.57	0.59	0.61	0.63	0.65
Vitamin premix^a^	1.00	1.00	1.00	1.00	1.00	1.00
Trace minerals^b^	0.10	0.10	0.08	0.06	0.04	0.02
Total	100.00	100.00	100.00	100.00	100.00	100.00
**Calculated nutrient level**						
Metabolizable Energy (MJ/kg)	12.54	12.54	12.54	12.54	12.54	12.54
Crude protein (%)	19.91	19.91	19.91	19.91	19.91	19.91
Calcium (%)	1.09	1.09	1.09	1.09	1.09	1.09
Available P (%)	0.46	0.46	0.46	0.46	0.46	0.46
Lysine (%)	1.37	1.37	1.37	1.37	1.37	1.37
Methionine (%)	0.40	0.40	0.40	0.40	0.40	0.40
Methionine + Cystine (%)	0.76	0.76	0.76	0.76	0.76	0.76

^a^The vitamin premix provided the following per kilogram of diet: vitamin A (retinyl acetate) 12,000 IU, vitamin D3 (cholecalciferol) 3,000 IU, vitamin E 20 mg, vitamin K3 1.3 mg, thiamine 2.2 mg, riboflavin 10 mg, Vitamin B3 10 mg, choline 400 mg, vitamin B5 50 mg, pyridoxine 4 mg, Biotin 0.40 mg, vitamin B11 1 mg, and vitamin B12 0.013 mg.

^b^The trace minerals provided the following per kilogram of diet: Cu 10 mg (cupric chloride), Fe 105 mg (ferrous sulfate), Zn 100 mg (Zinc sulfate), Mn 98 mg (manganous sulfate), Se 0.9 mg (sodium selenite), and I (calcium iodate) 0.575 mg.

Ten chickens were housed in each cage (length × width × height = 100 × 100 × 40 cm). 1 week before feeding, the room temperature was kept at 34 ± 1°C, and gradually decreased by 1°C per day until 24 ± 1°C. The relative humidity of the room was controlled at 50–60%. The illumination program used was 24L: 0D during the whole feeding trial. All chickens were having free access to mash feed and water in the morning and evening, respectively, and cleaned in the morning to maintain a good feeding environment in the house.

### Sample collection

During the trial, feed intake (FI) was recorded daily on a cage basis, whereas body weight gain (BWG) was monitored by weighting the birds at the beginning of the experiment and subsequently on day 56 (morning of slaughter). Average daily gain (ADG), average daily feed intake (ADFI), and feed conversion ratio (feed to gain ratio, F/G) were calculated. The calculation formula is as follows:


$$\begin{array}{l} \text{ADG}\left(\text{g}/\text{d}\right)=\text{BWG}\left(\text{g}\right)/56\text{d};\text{ADFI}\left(\text{g}/\text{d}\right)=\text{FI}\left(\text{g}\right)/56\text{d};\\ \text{ F}/\text{G}=\text{ADFI}/\text{ADG} \end{array}$$

During days 52 to 54, the broilers were fed an experimental diet and complete excreta were collected for 3 days (21). Excreta were collected thrice per day (07:00, 12:00, and 19:00 h) from each fecal tray under cages, weighed the weight of chicken excreta, mixed evenly, collected about 100 g of chicken excreta samples, and stored at −20°C. The excreta samples were dried for about 48 h in an oven at 65°C. The dried excreta was allowed to equilibrate to atmospheric conditions for 24 h before being weighed. Feed and excreta samples were then ground through a 0.425 mm screen and kept for further analysis.

At the age of 56 days, one chicken per replicate group was randomly selected and weighed after fasting for 12 h. Blood samples were collected through the vein wing into a vacutainer tube without anticoagulant. The serum was collected by centrifugation (3,000 g, 10 min, 4°C), and stored at −20°C for further analysis. The duodenum, jejunum, and ileum were, respectively, taken from the middle 3–4 cm segment, gently rinsed the intestinal contents with phosphate-buffered saline (PBS) solution, then quickly immersed the samples in a 4% paraformaldehyde fixed solution, and stored at 4°C for 24 h for later use. Samples of the duodenal and jejunal mucosal were quickly put into liquid nitrogen and stored at −80°C for further analysis.

### Chemical analysis

Feed and excreta samples were analyzed [AOAC (22), 2005] for dry matter (DM) (method 930.15), crude protein (CP) (N^*^6.25; method 984.13), and crude fat (CF) (method 920.39). Calcium and phosphorus determination was carried out according to the method of David et al. (23). The levels of serum endotoxin and D-lactate and serum diamine oxidase (DAO) activity were determined by assay kits (Shanghai Enzyme-linked Biotechnology Co., Ltd., Shanghai, China).

### Intestinal morphology

Samples of duodenum, jejunum, and ileum were tissue fixed in 4% paraformaldehyde, embedded in paraffin, sectioned longitudinally at 4 μm, and mounted on microscope slides. The sections were dewaxed for several times with xylene, rehydrated in alcohol, and stained with Haematoxylin/Eosin (H&E) (24). The villus height (VH) and crypt depth (CD) were measured using a microscope with VistarImage software (Olympus, Japan), and the VH to CD ratio (V/C) was calculated.

### Real-time PCR

Samples of the duodenal and jejunal mucosal were homogenized with an appropriate amount of TransZol. Total RNA was extracted from the duodenal and jejunal mucosal according to the instructions of TransZol Up Plus RNA kit. Then, the RNA was reverse transcribed into cDNA according to the instructions of the cDNA Synthesis SuperMix kit. Finally, real-time qPCR was performed on a CFX Connect Real-Time PCR Detect System (Bio-Rad, California, USA) according to the instructions of PerfectStart^TM^ Green qPCR SuperMix kit. The above kits were purchased from Beijing TransGen Biotech Co., Ltd. (Beijing, China). The primer sequences used in this study, as shown in Table 4, were designed and synthesized by Shanghai Generay Biotechnology Co., Ltd. (Shanghai, China). β-actin acts as the normalization of targeted genes. The 2^−ΔΔCt^ method was used to calculate the relative mRNA expression.

**Table 4:** Information on target genes and primers.

**Genes**	**Accession no**.	**Primer sequences (5^′^to 3^′^direction)**
Occludin	NM_205128.1	Forward: GGTCCCAGTAGATGTTGGCT Reverse: CCTCATCGTCATCCTGCTCT
Claudin-1	NM_001013611.2	Forward: CCAAGAAACAACCACCAGCA Reverse: TACAGCCCTTGGCCAATACA
ZO-1^a^	XM_040680632.1	Forward: ACTTGTAGCACCATCTGCCT Reverse: GAGCTCACAAGCTACGCAAA
β-actin	NM_205518.1	Forward: AAAGCCATGCCAATCTCGTC Reverse: ATCAGCAAGCAGGAGTACGA

^a^ZO-1, Zonula occludent1.

### Statistical analysis

Statistical analysis of all data was used for SPSS 25.0 version software (SPSS INC., Chicago, USA). Differences between UCCTM1000 group and CCTM1000 group were analyzed by two-tailed unpaired *t*-test, ^*^ *P* < 0.05 for the UCCTM1000 group vs. the CCTM1000 group. The CCTM groups were analyzed by one-way analysis of variance (ANOVA) followed by Duncan's *post-hoc* tests. Data were expressed as mean value ± standard error (SE), and significance was set at *P* < 0.05.

## Results

### Growth performance

**Figure 1:**
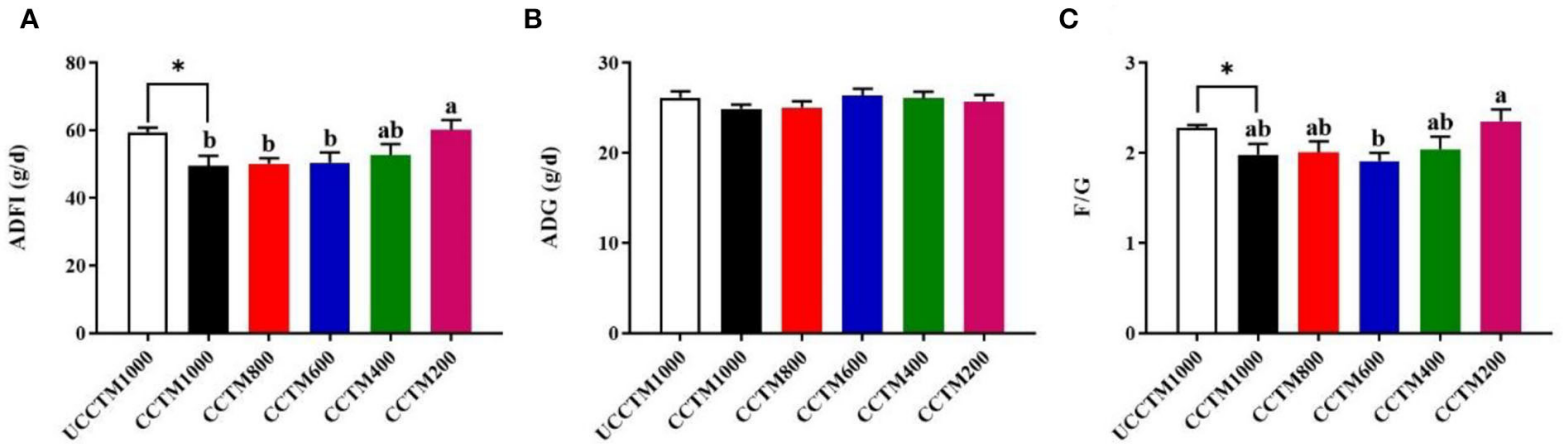
Effects of dietary coated complex trace minerals (TMs) on the growth performance of yellow-feathered broilers. **(A–C)** The 1 to 56 days of age average daily feed intake (ADFI), average daily gain (ADG), and feed to gain ratio (F/G) of yellow-feathered broilers. Results are mean value ± standard error (SE); *n* = 6 for each group. *P < 0.05 for the UCCTM1000 group vs. The CCTM1000 group. Mean values with different small letters denote significant differences among CCTM groups (*P* < 0.05). UCCTM1000, supplemented with 1,000 mg/kg uncoated complex trace minerals; CCTE1000, supplemented with 1,000 mg/kg of coated complex trace minerals; CCTE800, supplemented with 800 mg/kg of coated complex trace minerals; CCTE600, supplemented with 600 mg/kg of coated complex trace minerals; CCTE400, supplemented with 400 mg/kg of coated complex trace minerals; CCTE200, supplemented with 200 mg/kg of coated complex trace minerals.

As shown in Figure 1, compared with the UCCTM1000 group, the ADFI and F/G significantly decreased in the CCTM1000 group (*P* < 0.05), but there was no significant difference in the ADG between the UCCTM1000 and CCTM1000 groups (*P* > 0.05). The ADFI had a decreased tendency with dietary coated complex TMs addition level increasing (*P* < 0.05), the F/G was lower in the CCTE600 group than that in the CCTE200 group (*P* < 0.05), and the ADG was not affected by dietary coated complex TMs addition level (*P* > 0.05).

### Nutrients' digestibility

**Figure 2:**
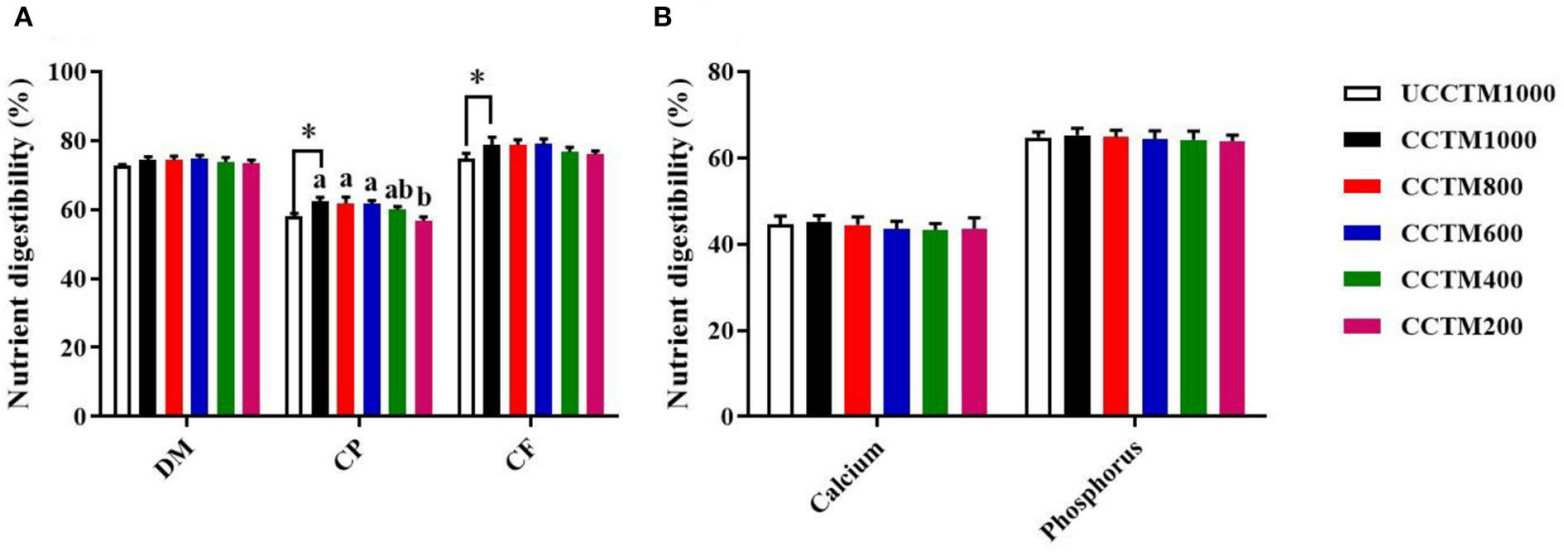
Effects of dietary coated complex TMs on nutrient digestibility of yellow-feathered broilers. The digestibility of **(A)** dry matter (DM), crude protein (CP), crude fat (CF), and **(B)** Calcium, and phosphorus were examined. Results are mean value ± standard error (SE); *n* = 6 for each group. *P < 0.05 for the UCCTM1000 group vs. the CCTM1000 group. Mean values with different small letters denote significant differences among CCTM groups (*P* < 0.05). UCCTM1000, supplemented with 1,000 mg/kg of uncoated complex trace minerals; CCTE1000, supplemented with 1,000 mg/kg of coated complex trace minerals; CCTE800, supplemented with 800 mg/kg of coated complex trace minerals; CCTE600, supplemented with 600 mg/kg of coated complex trace minerals; CCTE400, supplemented with 400 mg/kg of coated complex trace minerals; CCTE200, supplemented with 200 mg/kg of coated complex trace minerals.

As shown in Figure 2, compared with the UCCTM1000 group, the digestibility of CP and CF significantly increased in the CCTM1000 group (*P* < 0.05). The digestibility of CP was higher in the CCTE600, CCTE800, and CCTE1000 groups than that in the CCTE200 group (*P* < 0.05), and the digestibility of DM was not affected by dietary coated complex TMs addition level (*P* > 0.05). The digestibility of calcium and phosphorus was not affected (*P* > 0.05) among treatment groups.

### Intestinal morphology

**Figure 3:**
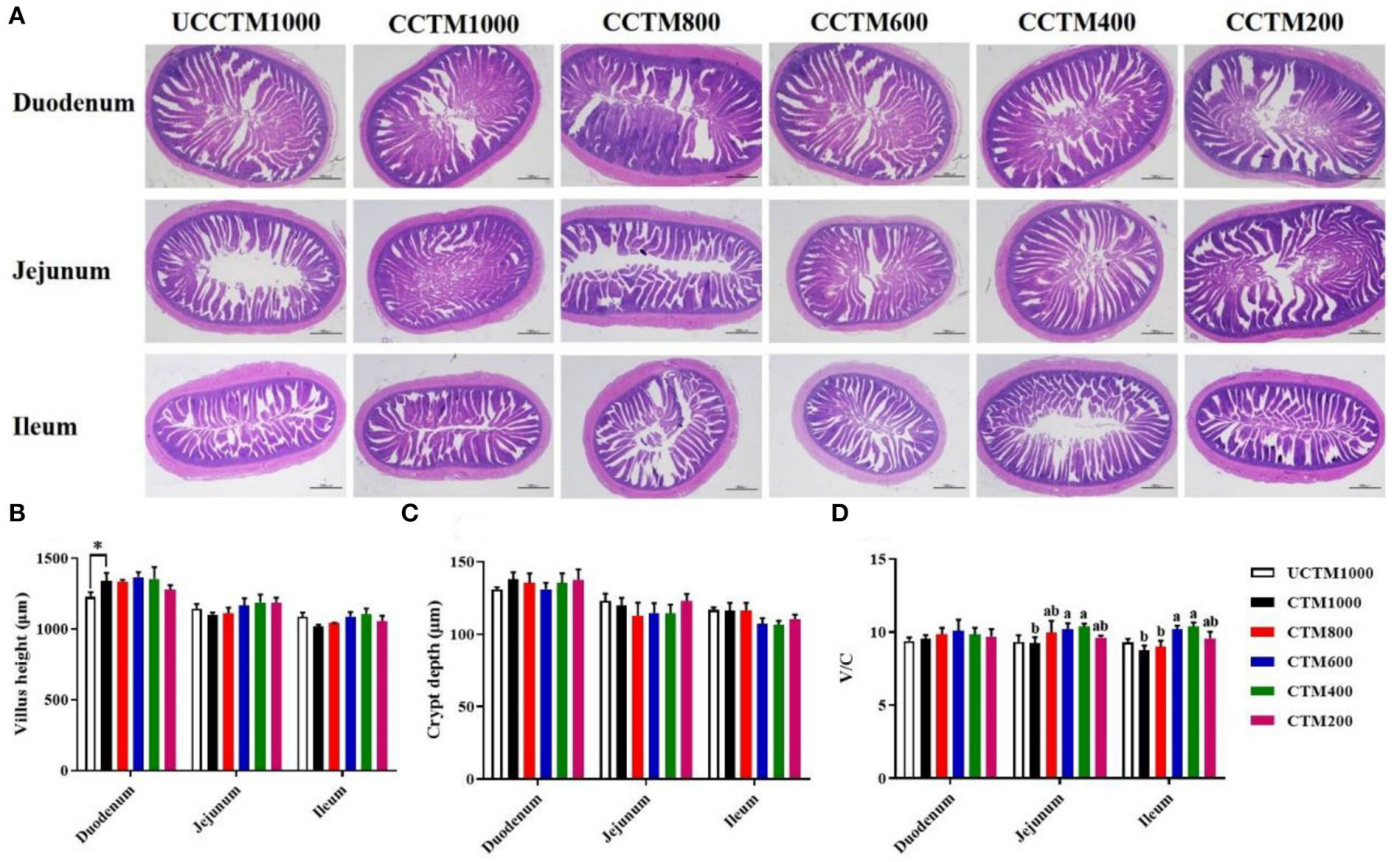
Effects of dietary coated complex TMs on small intestinal morphology of yellow-feathered broilers. **(A)** Representative hematoxylin/eosin (H&E) staining was taken at a magnification of x 20 and **(B)** villus height, **(C)** cry depth, and **(D)** ratio of villus height (VH) to crypt depth (CD) were measured. Scale bar = 1,000 μm. Results are mean value ± SE; *n* = 6 for each group. *P < 0.05 for the UCCTM1000 group vs. the CCTM1000 group. Mean values with different small letters denote significant differences among CCTM groups (*P* < 0.05). UCCTM1000, supplemented with 1,000 mg/kg of uncoated complex trace minerals; CCTE1000, supplemented with 1,000 mg/kg of coated complex trace minerals; CCTE800, supplemented with 800 mg/kg of coated complex trace minerals; CCTE600, supplemented with 600 mg/kg of coated complex trace minerals; CCTE400, supplemented with 400 mg/kg of coated complex trace minerals; CCTE200, supplemented with 200 mg/kg of coated complex trace minerals.

As shown in Figure 3, the intestinal tissue section images (Figure 3A) showed that the villi of the duodenum, jejunum, and ileum of CCTE400 and CCTE600 groups broilers did not show obvious fracture and fragmentation injury compared with the other groups. Compared with the UCCTM1000 group, the VH of the duodenum significantly increased in the CCTM1000 group (*P* < 0.05). The V/C of jejunum and ileum in the CCTM400 and CCTM600 groups were higher (*P* < 0.05) than that in the CCTM1000 group. The VH and CD of duodenum, jejunum, and ileum were not affected (*P* > 0.05) among treatment groups.

### Intestinal permeability

**Figure 4:**
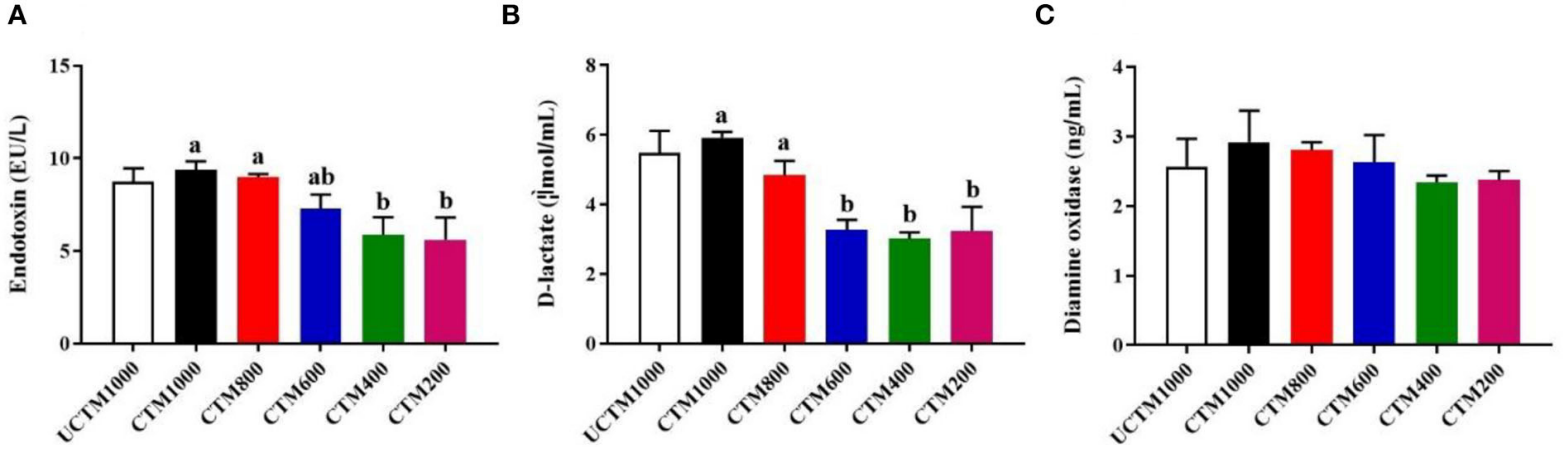
Effects of dietary coated complex TMs on intestinal permeability of yellow-feathered broilers. Serum **(A)** endotoxin concentration, **(B)** D-lactate level, and **(C)** diamine oxidase (DAO) activity were measured. Results are mean value ± SE; *n* = 6 for each group. Mean values with different small letters denote significant differences among CCTM groups (*P* < 0.05). UCCTM1000, supplemented with 1,000 mg/kg uncoated complex trace minerals; CCTE1000, supplemented with 1,000 mg/kg of coated complex trace minerals; CCTE800, supplemented with 800 mg/kg of coated complex trace minerals; CCTE600, supplemented with 600 mg/kg of coated complex trace minerals; CCTE400, supplemented with 400 mg/kg of coated complex trace minerals; CCTE200, supplemented with 200 mg/kg of coated complex trace minerals.

As shown in Figure 4, the serum levels of endotoxin and D-lactate were significantly increased with dietary coated complex TMs addition level (*P* < 0.05). The serum activity of DAO was not affected (*P* > 0.05) among treatment groups.

### Relative mRNA expressions of occludin, claudin-1, and ZO-1

**Figure 5:**
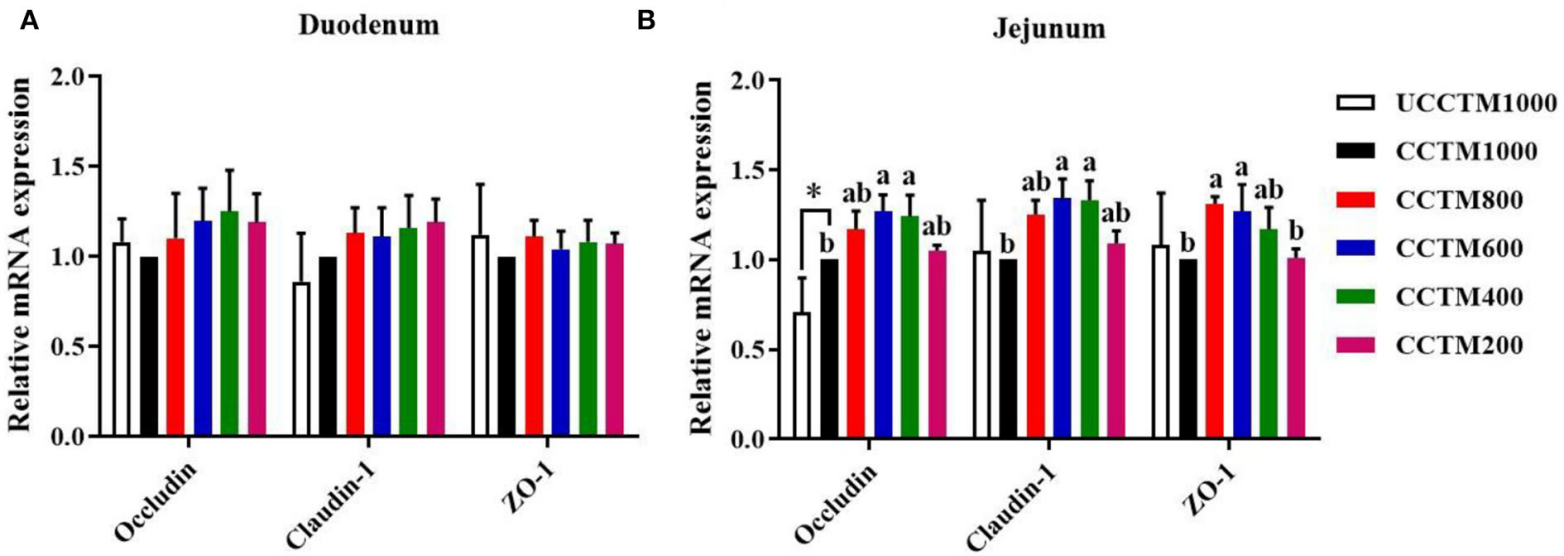
Effects of dietary coated complex TMs on the expression of tight junction protein mRNA in the duodenal and jejunal mucosa of yellow-feathered broilers. RT-PCR quantification of occludin, claudin-1, and zonula occludens-1 in **(A)** duodenal and **(B)** jejunal mucosa. Results are mean value ± SE; *n* = 6 for each group. *P < 0.05 for the UCCTM1000 group vs. the CCTM1000 group. Mean values with different small letters denote significant differences among CCTM groups (*P* < 0.05). UCCTM1000, supplemented with 1,000 mg/kg of uncoated complex trace minerals; CCTE1000, supplemented with 1,000 mg/kg of coated complex trace minerals; CCTE800, supplemented with 800 mg/kg of coated complex trace minerals; CCTE600, supplemented with 600 mg/kg of coated complex trace minerals; CCTE400, supplemented with 400 mg/kg of coated complex trace minerals; CCTE200, supplemented with 200 mg/kg of coated complex trace minerals.

As shown in Figure 5, the mRNA expression levels of occludin, claudin-1, and ZO-1 in duodenal mucosa were not affected (*P* > 0.05) among treatment groups. Compared with the UCCTM1000 group, the mRNA expression level of occludin in jejunal mucosa significantly increased (*P* < 0.05) in the CCTM1000 group. The mRNA expression levels of claudin-1 and ZO-1 in the CCTM600 group were higher (*P* < 0.05) than that in the CCTM1000 group.

## Discussion

### Growth performance and nutrient digestibility

The present study suggested that diets supplemented with 1,000 mg/kg of coated TMs had reduced F/G than the same level of uncoated TMs. Yin et al. (25) reported that coated TMs significantly improved the F/G than the uncoated ones. It is possible that coated TMs have higher digestibility, which not only reduces the complexation of TMs and anti-nutritional factors in the diets but also the antagonism between TMs was alleviated (26). Despite the fact that there is little information about the effect of coated TMs on broiler performance, it is possible that controlling the release of trace metals in the gastrointestinal tract could improve the efficacy of ion metal absorption (27). Previous reports have found that coating technology has become a performance enhancer due to its high bioavailability (20, 27, 28). In this study, the F/G of yellow-feather broilers fed 600 mg/kg of coated complex TMs in the diets was the lowest. Yin et al. reported that the F/G of ducks fed 500 mg/kg coated TMs in the diets was the lowest (28). Rao et al. (29) reported that the F/G was decreased by supplemented with low levels of organic TMs of their recommendations compared with those supplemented with 100% inorganic TMs. The cause behind the observed decrease in F/G associated with feeding coated TMs appears to be improved mineral utilization. Alternatively, it has been reported that the positive effect of coated TMs supplementation on broiler performance might be associated with the improvement of appetite and altered growth hormone production (20, 30).

The apparent digestibility of CP in this study was slightly lower compared with the values reported in other studies (31), likely due to the composition of the diet. Previous studies have shown that the fat to carbohydrate ratio (F/C) is the main factor affecting the digestibility of nutrients (32). Alimohamady et al. found that diets supplemented with inorganic zinc sulfate decreased the digestibility of crude protein (33). In this study, diets supplemented with 1,000 mg/kg of coated TMs could enhance the CP and CF digestibility when compared to the same level of uncoated TMs. The main functions of TMs are to be part of a host of coenzymes for various biological processes (34, 35). In addition, previous studies have shown that TMs such as zinc, iron, and manganese play a crucial role in the digestive enzyme activity of pancreatic tissue and the small intestine (30). Bile can promote fat digestion (36), while copper and zinc can affect bile secretion (37). Therefore, it is possible that dietary coated TMs may improve the growth of broilers by stimulating the enzyme activity of pancreatic tissue and small intestine and bile secretion involved in CP and CF digestibility. In the current study, the CP digestibility was greatly improved with increasing dietary coated complex TMs level, which is consistent with the Yin et al. report on poultry (28). The CF digestibility was not affected by the increasingly dietary coated TMs levels in this study, which are consistent with the previously demonstrated study in poultry (28, 38). But Wu et al. (9) reported that CF digestibility was increased by increasing dietary copper level. So far, the mechanism for coated TMs affecting the nutrient digestibility of poultry is still unclear and needs further study.

### Intestinal development

Intestinal morphology is directly related to the ability to digest and absorb nutrients of poultry (39), higher VH and lower CD make the intestinal digestion and absorption of nutrients stronger (40). In this study, diets supplemented with 1,000 mg/kg of coated TMs could enhance the VH in the duodenum more than the same level of uncoated TMs. The results of Shen et al. suggested that VH in the duodenum of piglets fed the coated zinc oxide was higher than that of those fed the zinc oxide (20). Previous studies have shown that TMs such as zinc, iron, and manganese are mainly absorbed in the duodenum (35). The reason behind the observed that supplementation coated TMs may improve intestinal development is increased mineral utilization efficiency. In this study, the broilers fed the coated complex TMs at the levels of 400 or 600 mg/kg showed the maximum V/C in the jejunum and ileum. In addition, the section results of HE staining in this experiment also showed that the villus of duodenum, jejunum, and ileum in coated 400 and 600 groups was orderly and complete compared with coated 200 and 1,000 groups. The results showed that supplementing with 400 or 600 coated complex TMs was beneficial to the growth and development of broiler small intestine tissue and promoted the digestion, absorption, and utilization of nutrients by increasing the absorption area of small intestine epithelium.

One of the main functions of the small intestine epithelium is acting as a barrier, which prevents antigens and pathogens from entering the mucosal tissues (41). The intestine epithelium injury may lead to increase intestinal permeability, which will promote the entering of toxic or allergenic substances from the gut into the body (42). The serum D-lactate, endotoxin content, and serum DAO activity were important indexes to assess intestinal barrier dysfunction, it says that intestinal barrier injury may lead to the increase of serum D-lactate content and DAO activity (43, 44). We observed that the levels of serum endotoxin and D-lactate were increased with increasing dietary coated complex TMs supplementation. It is possible that coated TMs have the characteristics of slow-release, which may increase the deposition of TMs in the body, and lead to intestinal tissue damage. Meanwhile, tight junctions (TJ) are complex structures composed of transmembrane proteins, which play a crucial role in maintaining the gut barrier function. The gene expression of tight junction proteins, including ZO-1, occludin, and claudin-1 protected intestinal barrier function (45, 46). In this study, diets supplemented with 1,000 mg/kg of coated TMs enhanced the expression levels of occludin mRNA in jejunal mucosa when compared to the same level of uncoated TMs. The addition of 600 mg/kg of coated Tes showed the maximum expression level of occludin, ZO-1, and claudin-1 mRNA in jejunal mucosa.

## Conclusion

In conclusion, the present study showed that coated complex TMs improved the growth and digestibility of crude protein and crude fat in yellow feather broilers. The addition of 600 mg/kg of coated complex TMs showed the minimum F/G and the maximum crude protein digestibility and intestine development of yellow-feathered broilers when compared with other treatments. This supplementation level of coated complex TMs could totally replace 1,000 mg/kg of uncoated complex TMs to further decrease the dose of TMs and raise economic benefit.

## Data availability statement

The datasets presented in this study can be found in online repositories. The names of the repository/repositories and accession number(s) can be found in the article/Supplementary material.

## Ethics statement

The animal study was reviewed and approved by Institutional Animal Care and Use Committee of Jiangxi Agricultural University (Nanchang, Jiangxi, China). The number of the Ethics Committee approved protocol for the animal study is JXAULL-2019-16. Written informed consent was obtained from the owners for the participation of their animals in this study.

## Author contributions

LX and MQ designed the overall study. CC wrote the manuscript. KO and HL provided helpful suggestions. ZX, YZ, and QY performed the experiments. All authors approved the submitted version.

## Funding

This work was financially supported by the Regional Project of the National Natural Science Foundation of China (32060760) and the Science and Technology Research Project of Jiangxi Provincial Department of Education (GJJ170260).

## Conflict of interest

Authors ZX and YZ were employed by the company Gongqingcheng Element Animal Nutrition Co., Ltd. The remaining authors declare that the research was conducted in the absence of any commercial or financial relationships that could be construed as a potential conflict of interest.

## Publisher's note

All claims expressed in this article are solely those of the authors and do not necessarily represent those of their affiliated organizations, or those of the publisher, the editors and the reviewers. Any product that may be evaluated in this article, or claim that may be made by its manufacturer, is not guaranteed or endorsed by the publisher.
