# Supplementary material for: Gastrointestinal digestibility insights of different levels of coated complex trace minerals supplementation on growth performance of yellow-feathered broilers
Source: Front Vet Sci. 2022 Sep 13;9:982699. doi: 10.3389/fvets.2022.982699 (PMC9513376; doi:10.3389/fvets.2022.982699)
Supplement: Supplementary file 1 [file Table_1.DOCX]

**Table S1.** The contents of trace minerals (uncoated and coated) of diets (mg/kg)

| 项目 | UCCTM 1000 | CCTM 1000 | *P*-value |
| --- | --- | --- | --- |
| Cu | 17.81±1.01 | 18.19±2.93 | 0.912 |
| Fe | 514.73±18.79 | 555.41±52.52 | 0.528 |
| Mn | 191.30±14.13 | 175.25±18.81 | 0.535 |
| Zn | 151.76±6.75 | 162.08±26.82 | 0.728 |
| Se | 0.42±0.03 | 0.38±0.03 | 0.493 |
